# Liberality in Medicine

**Published:** 1894-09

**Authors:** C. H. Oakes

**Affiliations:** Clinton, Mass.


### LIBERALITY IN MEDICINE.

C. H. Oakes, M. D., Clinton, Mass.

From time to time, and in some quarters with growing frequency, there is expressed a feeling that a more liberal spirit pervades the profession of medicine; that, in fact, the two schools are approaching and fraternizing in no small degree, and that, as a probable and altogether desirable result they will, at no distant day, in ecstatic union merged, constitute one incandescent mass of scientific liberality devoted solely to the "stamping out" process, the filling out of the never-to-be-forgotten whiskey or Morphine prescription-blank, and to the interests of the "dear people" in general. A liberal and paternal purpose for the profession, truly!

But it is not, on the whole, more than the *facts* of the last seventy-five years, together with the facts of to-day will warrant when any one adopts the above optimistic view. A careful study of the situation and the points involved, will, I think, demonstrate the fallacy of such conclusion.

And first, the situation embraces two radically different views of humanity and its ailments—the one school looking upon, almost exclusively, the physical or material side of humanity, considering it from a mechanical or chemical standpoint, and regarding a diseased human being as a disabled machine, to be repaired materially by very material means—more latterly by the various mechanical expedients of cutting, sawing, and filing, and by the administration, at odd intervals, of the odds and ends of crude drugs crudely combined—not because the more enlightened practitioners of that school believe those substances to be of value, but because their patients have for ages been accustomed to their use, and taught that their ills require them.

Thus, fully aware of the uncertainty of its methods of treatment—even to admitted failure in therapeutics, that school is to-day engaged in warfare upon the supposed causes of disease—always material—and hoping thereby to show reason why sentence should not be pronounced upon it—a sentence in accordance with its own confessions, and justly consigning it to the laboratory of some medical "old curiosity shop" which, in the fullness of time, may be the result of a general and all-pervading system of "medical legislation."

Such is the one, the allopathic school of to-day.

Obviously its approach to the more modern or homœopathic wing of the profession is not causing the axles of its ancient chariot to smoke with the fury of its coming.

Turning now to the homœopathic school for indications of the alleged process of amalgamation and of relinquishment of its distinctive doctrines and practices, what is the condition presented? Simply what any one versed in the homœopathic philosophy would expect to find—a rigid adherence to the natural law expounded by Hahnemann.

Were it otherwise there could be neither shred nor symptom of Homœopathy in existence, for any intentional departure from the above law, in the medicinal treatment of the sick, implies indefinite departure as occasion seems to demand, and therefore total relinquishment. Hence, the injury wrought by those of imperfect knowledge of homœopathic methods, the superficial dabblers—the traders upon a name, who have given color to the allopathic boast that there are, in practice, no real homœopaths ("none righteous, no not one").

"The two schools are approaching," thus becomes the plausible logic of newpapers, the superficialists above mentioned, and of many others who know nothing of Homœopathy.

Liberality in medicine indeed! What does it signify? Abandonment of a law of nature for the *more liberal use* of—

what? A more liberal use of which one of the sick-making drugs? Opium, first and chiefest of allopathic weapons, with a yearly importation of more than a billion allopathic doses? Calomel, Quinine, the Bromides—or the brain-compelling coal-tar derivatives?

Whiskey, the "favorite prescription" of the time-serving practitioner, who, being "his brother's keeper," dares to fling wide the gate to ruin by establishing an appetite for drink? Does it mean a more liberal use of the lancet—to the abandonment of which the followers of allopathy still "point with pride" as a shining and thoroughly isolated specimen of progress—is it the adoption of any or all of these condemned but still common agents that is to help the new school to approach and assist the old in the gentle gastronomic feat of swallowing each other?

Let homœopathic teachers and homœopathic physicians everywhere take pains to disabuse the public of the silly notion that has become so prevalent—the approach of the two schools. Not only should we do this, but we must encourage the missionary spirit that is properly a part of our philosophy.

No homœopathic physician is doing his whole duty to the world and to the cause of true healing unless he endeavors faithfully to place before the public the reasons for our therapeutic methods.

The people have a right to be and should be instructed; and professional reticence upon this matter is responsible for much delay in the adoption of rational means of cure.

